# MinION nanopore sequencing of an influenza genome

**DOI:** 10.3389/fmicb.2015.00766

**Published:** 2015-08-18

**Authors:** Jing Wang, Nicole E. Moore, Yi-Mo Deng, David A. Eccles, Richard J. Hall

**Affiliations:** ^1^Institute of Environmental Science and Research, National Centre for Biosecurity and Infectious Disease, Upper Hutt, New Zealand; ^2^WHO Collaborating Centre for Reference and Research on Influenza, Victorian Infectious Diseases Reference Laboratory, Peter Doherty Institute for Infection and Immunity, Melbourne, VIC, Australia

**Keywords:** influenza, virus, nanopore sequencing, MinION, whole genome sequencing

## Abstract

Influenza epidemics and pandemics have significant impacts on economies, morbidity and mortality worldwide. The ability to rapidly and accurately sequence influenza viruses is instrumental in the prevention and mitigation of influenza. All eight influenza genes from an influenza A virus were amplified by PCR simultaneously and then subjected to sequencing on a MinION nanopore sequencer. A complete influenza virus genome was obtained that shared greater than 99% identity with sequence data obtained from Illumina MiSeq and traditional Sanger-sequencing. The laboratory infrastructure and computing resources used to perform this experiment on the MinION nanopore sequencer would be available in most molecular laboratories around the world. Using this system, the concept of portability, and thus sequencing influenza viruses in the clinic or field is now tenable.

## Introduction

Next-generation sequencing technologies (NGS) are now capable of providing a whole genome sequence for most organisms (Vernikos et al., 2015). As such technologies have become more trusted, they are being applied with increasing frequency in clinical microbiology laboratories to detect and characterize pathogens; replacing the incumbent Sanger-sequencing methods (Bertelli and Greub, 2013; Quinones-Mateu et al., 2014). Sanger-sequencing has certainly been a reliable and robust method which has served molecular biology well for over three decades (McGinn and Gut, 2013). However, it is labor-intensive, slow, and not easily adapted for processing large genomes or large numbers of samples.

The characterisation of influenza viruses has certainly benefitted from whole genome sequencing (WGS) using Sanger-sequencing, providing a tool for surveillance of the highly dynamic genomes of influenza viruses (Ghedin et al., 2005). Such data can assist in vaccine development, detection of anti-viral drug resistance, or the identification of new reassortant influenza viruses. These advances may help to mitigate the morbidity and mortality of influenza pandemics or seasonal epidemics. Latterly, NGS platforms such as the Illumina MiSeq and Life Technologies Ion Torrent have received much acclaim about their ability to provide WGS data for influenza, potentially within 2–3 days of the receipt of a sample (Van den Hoecke et al., 2015). Such methods often use amplicon sequencing, where each of the eight gene segments are amplified individually or using generic primers (Van den Hoecke et al., 2015). Primers targeting the highly conserved 5′ and 3′ termini of each gene segment allow for simultaneous amplification of all segments within a single PCR reaction (Zhou et al., 2009). The use of a specific PCR avoids the issue of concomitant sequencing of host RNA (or DNA) that can occur in a metagenomic approach (Nakamura et al., 2009; Hall et al., 2014; Li et al., 2015). It then becomes possible to obtain WGS for influenza viruses that are present at low concentrations in a sample, as is commonly observed for clinical material.

However, there are also difficulties that attend the use of NGS for sequencing influenza. Such technologies require specialized instrumentation and reagents that are likely to be beyond the remit of diagnostic or regional reference laboratories. Furthermore, although the cost of NGS is decreasing, it is still prohibitive unless large numbers of samples are to be processed. There is clearly a place for a low-cost and portable NGS instrument that can be deployed more widely.

The MinION nanopore sequencing device has the potential to be the world’s first mobile sequencer (Quick et al., 2014). It is a small device (10 cm × 2 cm × 3.3 cm; approximately 90 g) and is powered by a computer USB port. Analysis of sequencing data is done in real time by an internet-connected laptop. Another unique feature of the device is the ability to generate very long reads of up to 60 kilobases (Madoui et al., 2015). It has been successfully applied for sequencing whole genomes of *E. coli*, poxviruses and lambda bacteriophage (Quick et al., 2014; Kilianski et al., 2015). It has also been applied to identify antibiotic resistance genes in repetitive regions of the genome of *Salmonella typhi* (Ashton et al., 2015). The MinION may therefore provide new opportunities in infectious disease diagnostics, for example, in the rapid sequencing of viral genomes in the clinic in response to the early phase of an influenza pandemic; or determination of ebola virus genotypes during an outbreak in a remote location. We show that whole genome sequence of a human (and animal) viral pathogen, the influenza virus, can be readily obtained using the MinION nanopore sequencer. These data are compared with an established Sanger-sequencing method and another NGS device, the Illumina MiSeq.

## Materials and Methods

### Nucleic Acid Extraction

The influenza A/New Zealand/316/2014 (H3N2) isolate was obtained from the culture collection at New Zealand’s World Health Organization National Influenza Centre, based at the Institute of Environmental Science and Research (ESR). RNA was extracted from 200 μL of MDCK-SIAT viral culture supernatant using the iPrep Purelink Virus kit (Life Technologies, Carlsbad, CA, USA), with elution into 50 μL of RT-PCR molecular grade water (Ambion, Austin, TX, USA).

### Sanger-sequencing

The eight influenza gene segments were first amplified using the RT-PCR protocol published by Deng et al. (2015). Briefly, cDNA was made using uni-12 primer [5′-AGCAAAAGCAGG] with ThermoScript RT-PCR system for first-strand cDNA synthesis kit (Invitrogen) as per the manufacturer’s instructions. Then 2 μl cDNA was added in 17 separate PCR reactions with Platinum Taq DNA polymerase high fidelity kit (Invitrogen) using gene specific primers that tagged with M13 universal sequences to the 5′ end. The PCR program used were as follows: 2 min at 94°C, then 35 cycles of 30 s at 94°C, 30 s at 55°C, and 1 min at 68°C, with a final extension at 68°C for 2 min. PCR amplicons were visualized by E-gel (Invitrogen), followed by ExoSAP IT (GE Healthcare) purification and used for sequencing with the forward and reverse M13 primers with Big Dye Terminator Reaction Mix (Applied Biosystems). The products were purified by Big Dye XTerminator Purification Kit (Applied Biosystems) and run on ABI 3500 XL Genetic Analyzer (Applied Biosystems). Sequencing results were analyzed using the DNASTAR Lasergene 9 package. The A/New Zealand/316/2014 full genome sequence is publically available from the GISAID EpiFlu database^1^ (accession numbers EPI587441-EPI587448).

### Illumina MiSeq High-throughput Sequencing

The eight influenza gene segments were amplified simultaneously in a single reaction using an RT-PCR with a single set of primers (MBTuni-12 [5′-ACGCGTGATCAGCAAAAGCAGG] and MBTuni-13 [5′-ACGCGTGATCAGTAGAAACAAGG]) (Zhou et al., 2009), including some modifications. Briefly, a 25 μL reaction was used for the Invitrogen Superscript III One-Step RT-PCR system with Platinum Taq DNA polymerase (Life Technologies) as per the manufacturer’s instructions, including 5 μL of template RNA. The thermocycling parameters were as follows: 60 min at 42°C, 2 min at 94°C, and then 5 cycles of 30 s at 94°C, 30 s at 45°C and 3 min at 68°C, followed by 35 cycles of 30 s at 94°C, 30 s at 57°C and 3 min at 68°C, with a final extension at 68°C for 2 min. PCR amplicons were visualized by agarose gel electrophoresis, followed by purification using the QIAquick PCR purification kit (Qiagen, Valencia, CA, USA) and DNA quantitation using the Qubit dsDNA BR assay (Life Technologies).

A sequencing library was then prepared using the Illumina TruSeq DNA Nano Library preparation kit (Illumina, San Diego, CA, USA), followed by sequencing of 250-bp paired-end reads on an Illumina MiSeq instrument (New Zealand Genomics Limited, Massey Genome Service, Palmerston North, New Zealand).

FastQC^2^ was used to check sequence data quality. Paired-end reads were then mapped in Bowtie2 (version 2.2.4) (Langmead and Salzberg, 2012) to a reference genome Influenza A/Christchurch/503/102 (H3N2), which was accessed from the Global Initiative on Sharing Avian Influenza Data EpiFlu™ Database^1^. Consensus sequence calling used SAMTools (Li et al., 2009). Data visualization was executed using Tablet (Milne et al., 2010) and Geneious R8 (Biomatters, New Zealand).

### MinION Library Preparation and Sequencing

This work was completed as part of the Oxford Nanopore Technologies (ONT) MinION early-access program. The Oxford Nanopore MinION Genomic DNA Sequencing kit (version 4) was used to prepare purified PCR amplicons (as for the Illumina MiSeq; see above) for sequencing according to the manufacturer’s instructions. Briefly, library preparation was as follows: 1 μg of purified PCR amplicon was diluted to 80 μL using RT-PCR molecular grade water (Ambion). The end repair process required the addition of 5 μL of Oxford Nanopore DNA CS and then use of the NEBNext End Repair module (New England Biolabs, Ipswich, MA, USA) by adding 10 μL of reaction buffer and 5 μL of enzyme, followed by a 30 min incubation at 25°C. The end repaired DNA was then purified using 100 μL Agencourt AMPure XP beads (Beckman Coulter Inc., Pasadena, CA, USA), as per the manufacturer’s instructions with elution into 25 μL of RT-PCR molecular grade water (Ambion). The NEBNext dA-tailing module (New England Biolabs) then required the addition of 3 μL of 10X reaction buffer and 2 μL of Klenow Fragment, followed by incubation of 30 min at 37°C.

8 μL nuclease-free water was added to 30 μL of the dA-tailed DNA, and then 10 μL adapter mix, 2 μL HP adapter and 50 μL Blunt/TA DNA ligase mastermix (New England Biolabs) were added in this order, and then incubated at 25°C for 10 min.

The ligated DNA was purified using His-tag Dynabeads. Beads were first prepared by washing in 200 μL of 1X ONT wash buffer twice, and then resuspended in 100 μL of 2X ONT wash buffer. The ligated DNA was mixed directly with the prepared beads and incubated at 25°C for 5 min. The beads were then washed twice again with 1X ONT wash buffer. 25 μL of ONT elution buffer was added to the beads with a 10 min incubation at 25°C. After removal of the beads, 6 μL of the eluate was combined with 141 μL EP buffer and 3 μL fuel mix, to provide the final sequencing library.

Before loading the library, the MinION device was connected to a computer and a flow cell (R7.3) inserted. A flow cell quality control check was run using the MinKNOW control software to assess pore activity. This was followed by equilibration of the flow cell with two aliquots of 150 μL priming mix (containing fuel mix and EP buffer), and observing a 10 min incubation between loading each aliquot.

The sequencing library was loaded into the flow cell. A 48-h sequencing protocol was selected on the MinION control software (version 0.46.1.9). The sequencing protocol was run for a total of 4 h.

### MinION Data Analysis

Raw sequence data was uploaded in real-time for base calling analysis using the cloud-based Metrichor workflow rx2.22-44717-dg-1.6.1-ch-1.6.3 rev 1.9 with the quality filter on. Reads in fastq format were then extracted from downloaded HDF5 format files (fast5) using poretools (Loman and Quinlan, 2014). Mapping used the same influenza reference genome as for the Illumina MiSeq data, but was performed using LASTAL (Frith et al., 2010) with the command line: lastal –s 2 –T 0 –Q 0 –a 1. SAMTools (0.1.19-44428cd) was used to convert the data into bam format. Tablet (1.14.04.10) and Geneious R8 (Biomatters, New Zealand) were used for visualization.

MinION sequence data has been deposited in the European Nucleotide Archive under project accession number PRJEB9812, available at www.ebi.ac.uk/ena.

## Results

**FIGURE 1:**
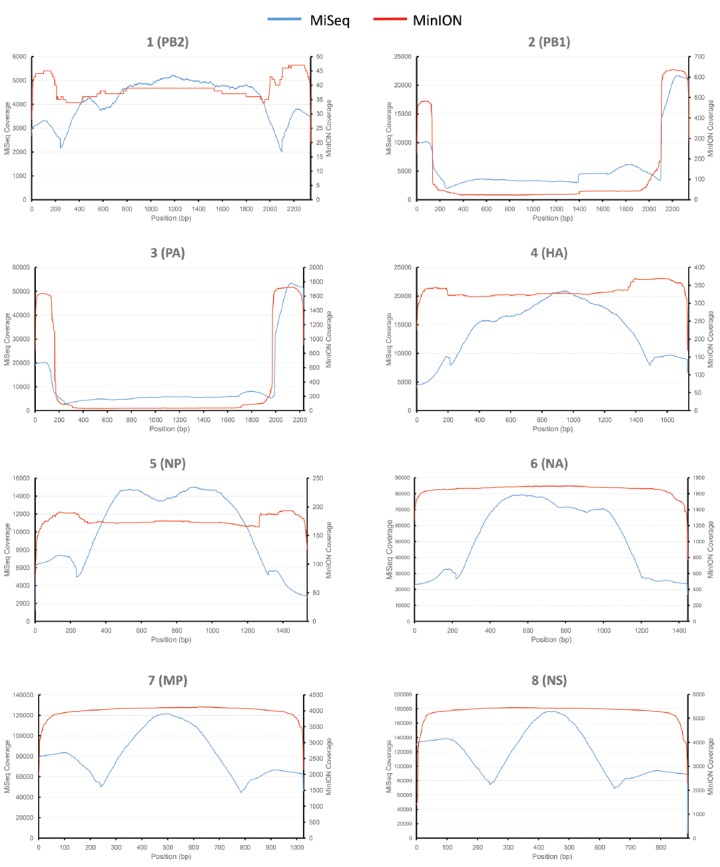
**Figure 1. Comparative analysis of influenza genome coverage, comparing the Illumina MiSeq, and the MinION nanopore sequencer.** Sequence coverage profiles for each influenza gene segment are plotted. The left-hand y-axis shows x-fold coverage for the Illumina MiSeq. The right-hand y-axis shows x-fold coverage for the MinION. The x-axis shows the position in the gene.

430 nanopores were available on the flowcell based on the Platform QC report generated by the control software (MinKNOW) prior to sequencing run commencement. In total, 118,052 sequence reads were produced in 4 h of run time (mean length 915 bp), with 17,108 reads categorized as high quality two-direction (2D) reads (mean length 955 bp). Sequence alignment to a prototypic reference genome was undertaken using the software package LASTAL, and full coverage of all influenza genes was achieved (Figure 1). Influenza sequence accounted for over 86.46% (14,793 reads) of the available 2D sequence data (Table 1).

**TABLE 1:** **Sequence read lengths and the proportions of reads assigned to the influenza genome**.

**Gene segment (Gene name)**	**1 (PB2)**	**2 (PB1)**	**3 (PA)**	**4 (HA)**	**5 (NP)**	**6 (NA)**	**7 (M)**	**8 (NS)**	**Total**
Gene length (bp)	2,341	2,341	2,233	1,733	1,537	1,448	1,027	890	13,550
No of 2D reads assigned	59	702	1,931	395	216	1,755	4,191	5,544	14,793
Percentage of total 2D reads	0.34%	4.10%	11.29%	2.31%	1.26%	10.26%	24.49%	32.41%	86.46%
Mean read length (bp)	1,243	245	490	1,498	1,259	1,418	1,045	913	–
Median read length (bp)	1,961	448	465	1,765	1,519	1,472	1,078	927	–
5th percentile (bp)	452	376	394	563	464	776	676	711	–
95th percentile (bp)	2,406	1,088	898	1,878	1,655	1,559	1,134	984	–
Minimum read length (bp)	313	206	211	369	343	223	171	283	–
Maximum read length (bp)	2,473	2,351	2,347	2,766	2,045	2,366	2,045	2,766	–

For comparison, single-tube PCR amplicons from the same influenza A (H3N2) influenza isolate were run on an Illumina MiSeq, producing 2,999,678 sequence reads of 250 bp in length. Bowtie2 was used to map sequence data to the same prototypic reference genome, resulting in 100% coverage, just as observed for the MinION nanopore sequencer (Figure 1). The coverage for the MiSeq was higher than for the MinION, due to the higher number of reads generated in the Illumina MiSeq run. The coverage for the MinION appears to be more even across the entire gene segment for HA, NA, NP, MP, and NS, as compared to coverage observed for the MiSeq.

**FIGURE 2:**
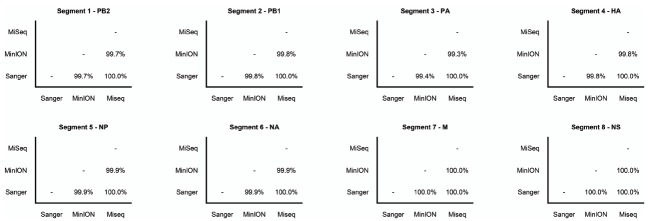
**Pairwise nucleotide sequence identity between influenza gene segments sequenced by Sanger-sequencing, Illumina MiSeq, and the MinION nanopore sequencer.** Gene names are also shown.

The Illumina MiSeq dataset showed 100% identity with the Sanger-sequence genome of the influenza (H3N2) virus (Figure 2). The MinION nanopore sequencer also showed high concordance with the Sanger-sequence data, having greater than 99% pairwise identity across the genome (Figure 2).

## Discussion

A whole genome sequence of an influenza virus was produced on the MinION nanopore sequencer using amplicon sequencing. All eight influenza gene segments from an influenza A (H3N2) virus were amplified simultaneously in a single PCR tube. This PCR method has previously been published, where primers target the conserved 12/13 base-pair viral RNA termini common to each influenza gene, and are conserved across all influenza A subtypes (Zhou et al., 2009). Amplicons were placed through the standard genomic DNA MinION workflow. The eight gene segments range in size from 0.89 to 2.3 kilobases, and amplification bias was apparent as reflected in the differing coverage for each gene segment (Figure 1) — an effect that has previously been documented (Van den Hoecke et al., 2015). Refined methods have now become available to limit this bias, which include adjustments of PCR primer concentrations, annealing temperature and elongation time (Van den Hoecke et al., 2015). The nucleotide discrepancies observed between the genome generated by the MinION as compared to the other methods are minimal but will still need to be resolved before the technique can be applied more widely. These discrepancies were distributed randomly throughout the genome and are most likely the result of sequencing error, as has been documented by others (Mikheyev and Tin, 2014; Quick et al., 2014; Kilianski et al., 2015). The per base accuracy of the MinION has been reported at between 65 and 80% (Kilianski et al., 2015). Therefore, discrepant nucleotides in the MinION-generated genome are not likely to represent genuine mutations that were missed by either Sanger-sequencing or the Illumina MiSeq. Point mutations can have significant phenotypic effects on influenza viruses, for example, in the neuraminidase gene they may confer resistance to antiviral drugs such as oseltamivir (Yen et al., 2006). It is therefore important that 100% identity with conventional sequencing approaches is attained before deployment in the clinic or field.

It is also unclear why the a proportion of the sequence reads generated in the MinION dataset are longer than the gene segment length, i.e. gene segment 4 (haemagglutinin) is 1,733 bp in length but records a maximum sequence read of 2,766 bp in the alignment (Table 1). This could be accounted for by the generation of chimeric amplicons during the PCR, where incomplete extension during the elongation phase of PCR provides heterologous templates for primers to bind to (Smyth et al., 2010). We could not provide direct evidence for this occurring but such an effect would not be able to be detected by a short-read NGS technology, even in the event where a chimeric junction is sequenced, as constraints of the mapping process would exclude such short reads from the final assembly. Similarly, Sanger-sequencing would be unlikely to reveal such chimera if they were rare enough (for example < 5%). However, an alternative explanation must be considered that an artifact of analysis or detection in the MinION instrument accounts for the apparent longer read lengths. Mapping of the MinION data using BWA (Li, 2013) was found to provide very similar results to LAST (data not shown), indicating that the mapping software is probably not the cause of such artifacts. *In silico* generation of chimeric sequence could occur if strand displacement at the nanopore is not recognized by the instrument during a run. This is speculation and further work will be required to resolve the reason for the increased apparent read lengths relative to the size of the original gene segments (data available from the European Nucleotide Archive; accession number PRJEB9812). It must be noted that the majority of MinION reads are of comparable length to their respective gene segments, i.e. 95% of reads mapping to HA are less than 1,878 bp, compared to the HA gene length of 1,733 bp (Table 1). The median read length for HA, NA, M, and NS is slightly longer than the gene segment length (Table 1), which could be due to sequencing of adaptors or insertions caused by sequencing error. Blast search of the unmapped portion of sequences failed to show any similarity with known sequences in genbank (data not shown).

The PA and PB1 genes appear to have higher coverage at the 5′ and 3′ termini, but lower coverage in the middle of the gene (Figure 1). This effect is observed in both MiSeq and MinION data. It is apparent to a lesser extent in the PB2 gene (probably masked by the low coverage in this gene). This is likely to have been caused by the presence of defective interfering viral particles, and very similar coverage profiles for NGS data have been noted before for PB2, PB1, and PA genes (Saira et al., 2013). Defective interfering virus particles of influenza have incomplete genomes, especially having truncated copies of PB2, PB1 and PA genes (Saira et al., 2013). This has resulted in an apparent lower median (and mean) read length for these genes in the MinION data (Table 1), which is an artifact of the short amplicons generated by the defective particles, rather than from the sequencing process itself.

The base-calling analysis for the MinION nanopore sequencer is an area of active development, as is the development of flowcells and associated enzymatic chemistries. The best quality sequence data are termed 2D reads. These are the consensus reads produced by comparing both the template and complementary strand sequences that are obtained when the double-helix is unwound and passed through the nanopore as a single-strand (Quick et al., 2014). Currently there are two technical aspects which affect the ability to produce 2D reads, those being the accuracy of measuring the electropotential across the nanopore, and the efficiency of the motor protein and hairpin adaptor binding. In the present study, 2D data account for 14.49% of the total reads. This figure is lower than previously published work by Quick et al (Quick et al., 2014) who report 22.6% 2D data when using the R7.3 flowcell. Although the accuracy of MinION sequence data is an area of active development and increases in data quality are anticipated (Bayley, 2015), this study shows the platform is already suitable for rapid influenza virus detection and provision of a draft genome. A 4 h run time for the MinION provides enough data to generate a reliable genome. But in order to attain 100% identity with other methods it will be necessary to increase sequence depth, most likely by an increase in run time.

Other single molecule sequencers, i.e., Helicos (Thompson and Steinmann, 2010) and Pacific Biosciences (Flusberg et al., 2010; McCarthy, 2010), require significant amounts of laboratory space, specialized equipment and large-scale computing resources. The MinION sequencer is small and portable. Run control and data analysis in this study were done in real-time using a moderate specification laptop (minimum hardware requirements: i7 CPU, 8 Gb RAM, and 128 GB solid state hard disk). This is in direct contrast to the high-end computing resources and expertise required to process short-read high throughput sequencing data for other platforms. The small footprint, potential for portability, and the use of common molecular biology equipment and reagents are attractive factors which show the MinION nanopore sequencer has potential in diagnostic applications. Furthermore, the short-run time of 4 h utilized in this study is a realistic timeframe for the production of clinically useful information. This study shows it is conceivable that investigators working in the field, for example, during an influenza pandemic or outbreak, could analyze samples on-site and draw initial conclusions on subtype, reassortants, pathogenicity and antiviral drug sensitivity (Barzon et al., 2013; Jones, 2015). The MinION nanopore sequencer can provide a useful method for diagnostic, research and reference laboratories to readily obtain full length influenza virus genomes.

### Conflict of Interest Statement

This work was done as part of the MinION Access Programme (MAP). The authors declare that the research was conducted in the absence of any commercial or financial relationships that could be construed as a potential conflict of interest.

## Footnotes

1 http://www.gisaid.org/

2 http://www.bioinformatics.babraham.ac.uk/projects/fastqc/
